# Knowledge and medication adherence of oral anticoagulant-taking patients in Vietnam

**DOI:** 10.1016/j.rpth.2023.100044

**Published:** 2023-01-11

**Authors:** Minh-Hoang Tran, Hoang Hai Nguyen, Quoc Khanh Mai, Hong Tham Pham

**Affiliations:** 1 Department of Pharmacy, Nhan Dan Gia Dinh Hospital, Ho Chi Minh City, Vietnam; 2 Department of Cardiology, Nhan Dan Gia Dinh Hospital, Ho Chi Minh City, Vietnam; 3 Department of Pharmacy, Nguyen Tat Thanh University, Ho Chi Minh City, Vietnam

**Keywords:** anticoagulants, knowledge, medication adherence, Southeast Asia, Vietnam

## Abstract

**Background:**

Oral anticoagulants for venous thromboembolism are not thoroughly managed in ambulatory settings in low-/middle-income countries, primarily because of patients’ neglect of medication knowledge and adherence.

**Objectives:**

This study was conducted to investigate patient knowledge, adherence, and the associated factors at a Vietnamese tertiary hospital, serving as a reference for educational programs in other local and regional health care facilities.

**Methods:**

A randomly recruited cross-sectional study was conducted on patients using vitamin K antagonists (VKA) or direct oral anticoagulants (DOAC). The primary and secondary outcomes were the knowledge score (in percent) and adherence to oral anticoagulants. Student’s *t*-tests or chi-squared tests were used to compare the crude differences in mean or proportion between patients taking VKA and DOAC. Regression models were conducted to adjust the potential confounders and determine factors associated with patient knowledge and adherence.

**Results:**

A total of 199 patients were included. After adjusting for potential confounders, patients receiving a DOAC had similar knowledge scores and levels of adherence compared with those taking VKA, with both groups being suboptimal. Previous counseling was associated with higher knowledge. Better medication knowledge, female sex, and no history of venous thromboembolism were associated with better adherence.

**Conclusion:**

Good knowledge and adherence of oral anticoagulant-taking patients remain limited in Southeast Asian clinical practice. Further studies in similar settings should examine the associations between these factors and the effectiveness of the regimens. More effective measures targeting patient knowledge and adherence should be implemented to optimize anticoagulation therapy, improve the patients’ outcomes, and mitigate the associated adverse effects.

## Essentials

• Patient knowledge and adherence to oral anticoagulants relates to outcomes in venous thromboembolism.
• Patient knowledge and adherence to oral anticoagulants were assessed in a middle-income country.
• Knowledge and adherence were similar for vitamin K antagonist and direct oral anticoagulant users.
• Knowledge was better with previous counseling; adherence was better in women or patients with good knowledge or no previous venous thromboembolism.

## 1 Introduction

Venous thromboembolism (VTE) is a hematologic disorder that can lead to serious conditions with high mortality and health expenditure consuming. The incidence rates of VTE in Asian populations can range from 8 to 20 events per 100,000 person-years [1], which could be higher in European or American populations [2]. The recurrence rate in Taiwan, a country in Asia, was more than 5% and was most observed during the first 12 months following a VTE event [1]. In some Asian settings, VTE-associated mortality rates could vary from 4.3% after admission and up to 14.9% within 6 months after discharge [1].

Anticoagulants have remained the primary regimens for prophylaxis and treatment of VTE. With the ability to block the coagulation pathways, these medications are accompanied by bleeding and bleeding-associated events [3]. Although the uses of intravenous and subcutaneous anticoagulants are strictly monitored in clinical settings to prevent these adverse events, the oral counterparts have not been thoroughly managed in ambulatory patients, which could lead to potential drug misuse, especially in low- and middle-income countries such as Vietnam. This could create additional burdens and challenges (poor health outcomes, increased cost, etc.) for the patients and health care systems because of unoptimized use of oral anticoagulants.

To improve the management of oral anticoagulants in ambulatory settings, targeting patients’ knowledge and adherence to their regimens can play a very important role within a patient-centered practice. In patients using warfarin, a vitamin K antagonist (VKA), better knowledge and adherence are associated with more optimal health outcomes [4]. However, in many clinical settings, existing evidence lacks the focus on the widely used direct oral anticoagulants (DOAC), possibly because of their advantageous pharmacologic characteristics and convenient use over VKA [5]. This neglect of DOAC may increase the likelihood of drug misuse and contradictorily worsen health outcomes despite the superior benefits of DOAC. Given the lack of evidence in Vietnam, this study was conducted to comprehensively assess and identify the associated factors of knowledge and adherence to both VKA and DOAC therapy at a local tertiary hospital, which serves as a reference to develop and implement new measures for managing oral anticoagulants in other local and regional health care facilities.

## 2 Methods

### 2.1 Study design and participants

A cross-sectional study was conducted at Nhan Dan Gia Dinh (NDGD) Hospital from 1 April to 30 June of 2019 (the second quarter of 2019). All patients having medical examinations at NDGD Hospital and undertaking oral anticoagulants were randomly recruited into the study using a sampling frame of ambulatory patients under the management of NDGD Hospital. Patients were eligible if they (1) were ≥18 years old, (2) were treated for more than 2 months with VKA (group 1) or DOAC (group 2) (exposure of interest), and (3) agreed to participate in the study. Patients were excluded if they (1) were pregnant or breastfeeding women, (2) were unable to comprehend the instructions to complete the questionnaire, or (3) had no caretakers in case they were unable to respond to the questionnaires. This study followed the Strengthening the Reporting of Observational Studies in Epidemiology (STROBE) Statement (Supplementary Checklist).

### 2.2 Study outcomes

The primary outcome was the patients’ knowledge score (in percent) of oral anticoagulants, which was measured using the Anticoagulant Knowledge Tool (AKT) scale [6]. The AKT includes 2 sections, with section A applicable to patients taking DOAC or VKA and section B specific to those in use of VKA. The secondary outcome was the proportion of patients’ adherence to oral anticoagulants, assessed using the Morisky Green Levine Scale (MGLS) [7]. The levels of adherence were defined as follows: (1) high (score of 0), (2) medium (score of 1 or 2), and (3) low (score of 3 or 4).

### 2.3 Reliability of AKT and MGLS questionnaires

The AKT and MGLS questionnaires were previously tested for reliability through a two-stage convenience-sampling pilot survey (*n* = 100) using a similar sampling frame from 5 January to 20 March of 2019. After removing questions number 5, 9, 12, and 13 owing to low values of corrected item-total correlation, the Cronbach alpha values of the AKT questionnaire in patients receiving VKA and DOAC were 0.713 and 0.705, in turn, whereas the corresponding value of the MGLS questionnaire was 0.701 with no additional issues, which were all acceptable to confirm the internal consistency reliability of these research instruments in this study [8]. A test-retest reliability was also conducted following the second stage (2 months after the first stage) of the pilot survey. The intraclass correlation coefficients (ICC) for the VKA and DOAC sets of questions in the AKT questionnaire were 0.721 and 0.742, respectively. The MGLS questionnaire had an ICC of 0.767. These have corroborated the reliability of the AKT and MGLS questionnaires over time in this study setting [9].

### 2.4 Sample size

The required sample size was calculated using the online calculator from the following website: http://powerandsamplesize.com/Calculators/, with type I error rate of 5%, power of 80%, and a sampling ratio of 1:1. Following our pilot survey, the mean AKT scores (in percent) of patients taking VKA and DOAC were 40.2% and 34.4%, respectively, with an overall estimated SD of 13.1%, giving a minimum sample size of 80 patients per group.

### 2.5 Assessment of covariates

The following covariates were included in this study as potential confounders: interviewed subjects (patients/caretakers), age, gender (male/female), education level (primary or lower/secondary or higher), occupational status (high/middle/low), previous counseling (yes/no), and previous VTE (yes/no).

### 2.6 Statistical analysis

Responses with missing data were excluded from the analyses. For continuous variables, demographic data were presented as medians with IQRs (Q1–Q3) and two-sample *t*-test was used to compare the crude mean differences between the 2 groups. A multiple linear regression model was conducted to adjust for potential confounders and identify the factors associated with patients’ knowledge of oral anticoagulants. For categorical variables, we presented data as frequencies with percentages and used the chi-squared test to compare the crude difference in proportions. We also used a multinomial logistic regression model to determine the adjusted effect and moderating factors of patients’ adherence (high, medium, and low) to their medications. In both cases, type II anova test was taken to confirm the factors’ effects on knowledge or adherence. All hypotheses were tested with a confidence level of 95%. We tested for interactions between the exposure and other potential confounding covariates. No statistical interactions were found (p_interaction_ > 0.05). The statistical analyses were performed using R (version 4.1.2, R Foundation for Statistical Computing).

### 2.7 Sensitivity analysis

Owing to the high-risk nature of patients with heart valve replacement, we conducted a sensitivity analysis that excluded these patients to further explore the impact of oral anticoagulants on medication knowledge and adherence. All settings from the primary analysis were reapplied to the multiple linear and multinomial logistic regressions to provide the adjusted results.

### 2.8 Ethics approval

This study was approved by the NDGD Hospital Ethics Committee, Ho Chi Minh City, Vietnam, under approval number 22-2019/NDGD-HDDD. The included patients all gave their informed consent.

## 3 Results

### 3.1 Patient characteristics

During the investigated period, a total of 240 subjects were invited, of which 41 were excluded because of incomplete responses to the AKT or MGLS questionnaires or declining to participate, giving 199 eligible observations for analysis (Supplementary Figure 1). There was no significant difference between the characteristics of those included and excluded (Supplementary Table 1).

Table 1 summarizes the characteristics of the patients being prescribed VKA or DOAC. Overall, the median age of all patients was 63 (55–70), of which patients taking DOAC were slightly older than those receiving VKA. All patients were Vietnamese-origin Asians. The proportions of men and women were similar in both VKA and DOAC groups. Although most patients undertook secondary education or higher (71.9%), most occupational status remained at a low level (76.4%). The prevalences of patients receiving previous medication counseling were consistently low (17.1%) in both VKA and DOAC groups.

**Table 1:** Characteristics of patients taking VKA and DOAC.

Variable	VKA (*n* = 117)	DOAC (*n* = 82)	Total (*n* = 199)
Interviewed subjects, *n* (%)			
Patients	104 (88.9)	68 (82.9)	172 (86.4)
Direct caretakers	13 (11.1)	14 (17.1)	27 (13.6)
Age, median (Q1–Q3)	60 (51–69)	67 (60.3–72.8)	63 (55–70)
Age category, *n* (%)			
18–65 y old	85 (72.6)	43 (52.4)	128 (64.3)
>65 y old	32 (27.4)	39 (47.6)	71 (35.7)
Gender, *n* (%)			
Male	51 (43.6)	44 (53.7)	95 (47.7)
Female	66 (56.4)	38 (46.3)	104 (52.3)
Asian race, *n* (%)	117 (100)	82 (100)	199 (100)
Education, *n* (%)			
Primary or lower	36 (30.8)	20 (24.4)	56 (28.1)
Secondary or higher	81 (69.2)	62 (75.6)	143 (71.9)
Occupational status, *n* (%)			
High	8 (6.8)	4 (4.9)	12 (6.0)
Middle	24 (20.5)	11 (13.4)	35 (17.6)
Low	85 (72.6)	67 (81.7)	152 (76.4)
Previous counseling, *n* (%)	20 (17.1)	14 (17.1)	34 (17.1)
Previous VTE, *n* (%)	9 (7.7)	13 (15.9)	22 (11.1)
Cardiovascular hospitalization, *n* (%)^a^			
Heart surgeries	64 (54.7)	3 (3.7)	67 (33.7)
Cardiovascular events	19 (16.2)	20 (24.4)	39 (19.6)
Abnormal bleeding/hypercoagulation	8 (6.8)	3 (3.7)	11 (5.5)
None	26 (22.2)	56 (68.3)	82 (41.2)
Indications for anticoagulants, *n* (%)^a^			
Heart valve replacement	69 (59.0)	0 (0.0)	69 (34.7)
Thrombosis	9 (7.7)	13 (15.9)	22 (11.1)
Valvular atrial fibrillation	31 (26.5)	7 (8.5)	38 (19.1)
Nonvalvular atrial fibrillation	34 (29.1)	59 (72.0)	93 (46.7)

DOAC, direct oral anticoagulant; VKA, vitamin K antagonist; VTE, venous thromboembolism.

a May not add up to 100% because of rounding.

### 3.2 Treatment with oral anticoagulants

Acenocoumarol was the most frequently used anticoagulant (58.8%), possibly because of its lower cost and broader indications compared with DOAC groups (eg, nonvalvular atrial fibrillation, thrombotic prophylaxis in patients having heart valve replacement surgeries, etc.) [5]. Dabigatran and rivaroxaban were the only available DOAC during the investigated period, accounting for 18.6% and 22.6% of the anticoagulants, respectively. Those taking DOAC were not recommended any routine monitoring, whereas all patients taking acenocoumarol had their international normalized ratios measured at baseline and once every 4 weeks.

### 3.3 Knowledge of oral anticoagulants

Patients’ knowledge of oral anticoagulants is shown in Table 2, with the detailed responses presented in Supplementary Table 2. In terms of general knowledge (applicable to both VKA and DOAC groups), the overall score of all the patients was 46.3%. Patients receiving VKA had a higher crude average score than those taking DOAC (mean difference, 7.5%; 95% CI, 2.6%–12.5%; *P* = .003). For the total knowledge (with an additional section assessing patients taking VKA), the overall score was 45.2%. There was also a similar trend to the general knowledge favoring the VKA group (mean difference, 5.6%; 95% CI, 1.1%–10.9%; *P* = .018).

**Table 2:** Knowledge of oral anticoagulants of the patients.

Knowledge score	VKA (*n* = 117)	DOAC (*n* = 82)	Total (*n* = 199)	*P* value^a^
General knowledge (section A)				
Mean	10.4	8.8	9.7	
Percentage (%)	49.4	41.9	46.3	.003
Total knowledge (section A + B)^b^				
Mean	13.8	8.8	11.7	
Percentage (%)	47.5	41.9	45.2	.018

DOAC, direct oral anticoagulant; VKA, vitamin K antagonist.

a Two-sample *t*-test between VKA and DOAC groups, with equal variance.

b Section B is only applicable to VKA group.

However, after adjusting for the potential confounders, the AKT scores of VKA and DOAC groups did not differ significantly (Table 3). Previous counseling was the only factor found to influence knowledge of oral anticoagulants. The average score of patients who were previously counseled was 11.1% higher than that of those who were not (95% CI, 5.6%–16.3%; *P* < .001). Age was another potential moderating factor (*P* = .063) because the older tended to have lower knowledge scores than younger patients, although this association was not statistically significant.

**Table 3:** Factors affecting patients’ knowledge of oral anticoagulants (in percent).

Variable	Effect	95% CI	*P* value^a^	
			Student’s *t*-test	*F*-test
Type of oral anticoagulants				0.124
VKA^b^	–	–	–	
DOAC	−3.3	−7.5 to 0.9	0.124	
Interviewed subjects				0.262
Patients^b^	–	–	–	
Direct caretakers	−3.6	−10.0 to 2.7	0.261	
Age	−0.2	−0.4 to below 0.1	0.063	0.063
Gender				0.720
Male^b^	–	–	–	
Female	0.7	−3.3 to 4.7	0.720	
Education				0.085
Primary or lower^b^	–	–	–	
Secondary or higher	3.7	−0.5 to 7.8	0.085	
Occupational status				0.367
High^b^	–	–	–	
Middle	−6.6	−15.8 to 2.6	0.158	
Low	−5.1	−13.7 to 3.4	0.236	
Previous counseling	11.1	5.9–16.3	<0.001	<0.001
Previous VTE	−2.6	−9.2 to 3.9	0.429	0.429

DOAC, direct oral anticoagulant; VKA, vitamin K antagonist; VTE, venous thromboembolism.

a Multiple linear regression.

b Reference level.

### 3.4 Medication adherence

Table 4 shows the overall and stratified (by types of oral anticoagulants) medication adherence of the participants, with the detailed responses presented in Supplementary Table 3. Medium or high levels of adherence were reported by most interviewed patients (over 90%). Similar patterns were observed when stratifying the patients by their prescribed medications of VKA or DOAC ($\chi_{\left(2\right)}^{2}=0.053$, *P* = .974).

**Table 4:** Patients’ medication adherence to oral anticoagulants.

Level of adherence	VKA (*n* = 117)	DOAC (*n* = 82)	Total (*n* = 199)	*P* value^a^
Medication adherence, *n* (%)				.974
High	44 (37.6)	31 (37.8)	75 (37.7)	
Medium	64 (54.7)	44 (53.7)	108 (54.3)	
Low	9 (7.7)	7 (8.5)	16 (8.0)	

DOAC, direct oral anticoagulant; VKA, vitamin K antagonist.

a Chi-squared test between VKA and DOAC groups.

Factors affecting the patients’ adherence to oral anticoagulants were knowledge of oral anticoagulants, gender, and history of VTE (Table 5). Higher knowledge of the medications is associated with a greater possibility of adherence (*P* < .05). Although there was no difference in terms of gender when comparing low to high adherence (odds ratio [OR], 1.17; 95% CI, 0.32–4.22; *P* = .812), female patients were found to have greater odds of high over medium adherence than male peers (OR, 0.46; 95% CI, 0.24–0.87; *P* = .018). History of VTE did not significantly alter the scale between medium and high adherence (OR, 2.02; 95% CI, 0.57–7.22; *P* = .278) but increased the risks of low over high adherence (OR, 8.57; 95% CI, 1.62–45.52; *P* = .012). Despite yielding significant results, occupational status and previous counseling were not statistically identified as moderating factors for oral anticoagulant adherence, possibly because of unstable estimates from the small sample size of patients with low adherence.

**Table 5:** Factors affecting patients’ medication adherence to oral anticoagulants.

Variable	Medium			Low			*P* value (LR test)
	OR	95% CI	*P* value^b^	OR	95% CI	*P* value^b^	
Type of oral anticoagulants							0.862
VKA^a^	–	–	–	–	–	–	
DOAC	0.88	0.44–1.76	0.716	0.73	0.21–2.49	.612	
Knowledge	0.05	below 0.01–0.49	0.011	<0.01	below 0.01–0.29	.013	.006
Interviewed subjects							.890
Patients^a^	–	–	–	–	–	–	
Direct caretakers	1.14	0.40–3.30	0.806	1.51	0.28–8.10	.629	
Age	0.99	0.96–1.02	0.346	1.00	0.95–1.06	.990	.596
Gender							.031
Male^a^	–	–	–	–	–	–	
Female	0.46	0.24–0.87	0.018	1.17	0.32–4.22	.812	
Education							.904
Primary or lower^a^	–	–	–	–	–	–	
Secondary or higher	0.89	0.47–1.79	0.751	1.12	0.33–3.87	.855	
Occupational status							.657
High^a^	–	–	–	–	–	–	
Middle	0.90	0.19–4.26	0.892	0.97 × 10^6^	(0.21–4.52) × 10^6^	<.001	
Low	0.71	0.17–2.97	0.641	0.98 × 10^6^	(0.18–5.22) × 10^6^	<.001	
Previous counseling	1.31	0.56–3.09	0.532	<0.01	–	<.001	.160
Previous VTE	2.02	0.57–7.22	0.278	8.57	1.62–45.52	.012	.039

Reference level for medication adherence was high.

DOAC, direct oral anticoagulant; LR test, likelihood-ratio test; OR, odds ratio; VKA, vitamin K antagonist; VTE, venous thromboembolism.

a Reference levels for the covariates.

b Wald test; multinomial logistic regression.

### 3.5 Sensitivity analysis

The results of the sensitivity analysis were summarized in Table 6. Similarly, the types of oral anticoagulants did not show any significant impacts on patients’ knowledge or adherence (*P* > .05), after adjusting for the potential confounders. Compared with the primary analysis, whereas previous counseling still affected patients’ knowledge (*P* = .007), only knowledge had an effect on medication adherence (*P* = .002).

**Table 6:** Sensitivity analysis after the exclusion of patients with heart valve replacement.

Variable	Knowledge			Medication adherence						
	Effect	95% CI	*P* value^a^	Medium			Low			*P* value (LR test)
				OR	95% CI	*P* value^b^	OR	95% CI	*P* value^b^	
Type of oral anticoagulants										.663
VKA^c^	–	–	–	–	–	–	–	–	–	
DOAC	−0.2	−5.8 to 5.4	0.942	1.04	0.48 to 2.26	.921	0.61	0.18–2.11	.435	

Effect and 95% CI on knowledge were presented in percent with 1 decimal place. Reference level for medication adherence was high; OR and 95% CI on medication adherence were presented with 2 decimal places.

DOAC, direct oral anticoagulant; LR test, likelihood-ratio test; OR, odds ratio; VKA, vitamin K antagonist.

a *t*-test; multiple linear regression; adjusted for interviewed subjects, age, sex, education, occupational status, previous counseling, previous venous thromboembolism.

b Wald test; multinomial logistic regression; adjusted for medication knowledge, interviewed subjects, age, sex, education, occupational status, previous counseling, previous venous thromboembolism.

c Reference level.

## 4 Discussion

Overall, our study observed a higher crude knowledge score in patients taking VKA compared with those with DOAC, but the patterns of confounder-adjusted knowledge and medication adherence were similar between the 2 groups. We found 1 factor affecting knowledge of oral anticoagulants to be previous counseling, whereas adherence to the medications was moderated by knowledge of the therapy, gender, and previous VTE.

In terms of the knowledge score, although the adjusted model showed no difference between the VKA and DOAC groups, the crude result was pretty comparable to the research of Obamiro et al. [10], where the AKT score of patients receiving VKA (73%) was significantly higher than those in use of DOAC (66%). This could be owing to a concerning profile of adverse effects and drug–drug/drug–food interactions of VKA, urging physicians to provide additional information and advice for their patients to optimize the regimen with VKA. By contrast, the lack of knowledge could cause drugs with safer profiles like DOAC to be much more harmful to patients, especially when access to antidotes of DOAC (idarucizumab, andexanet alfa) [11] is limited in low- and middle-income countries such as Vietnam.

Although previous counseling and age seem to be potential moderating factors for patients’ knowledge, we could not find a significant relationship between the latter and knowledge of oral anticoagulants, partially because of the limited number of participants in our study. A larger sample size could increase the probability of detecting this association, as suggested by the previous findings [10,12,13].

Regarding adherence to treatment, we only found a pretty low proportion of good adherence to oral anticoagulants, which was much lower compared with the adherence rate of previous findings (over 50%) [10,14]. The reason for this discrepancy could possibly be the difference in the scales to determine medication adherence because most publications used the 8-item Morisky Medication Adherence Scale (MMAS-8), an improved version of the MGLS that we used in this study [15]. However, a quick survey in our hospital has shown the patients’ preferences for the MGLS over the MMAS-8 because the former was much shorter and easier to understand, which partly justified why we used the MGLS in our study. Despite using different scales, the distributions in adherence levels between the VKA and DOAC groups did not differ significantly, reflecting consistency with other studies [10,14]. This could partially support the use of the cost-saving MGLS in low- or middle-income settings rather than the much more expensive MMAS-8.

Noticeably, we also identified 3 factors that were associated with the medium or low over high level of adherence to oral anticoagulants. Educational measures to improve knowledge of medication are likely to boost adherence, regardless of patients’ current level. Counseling sections should also target male patients with medium levels of adherence and those having low adherence with prior VTE because they have the highest likelihood of benefiting from educational programs. Moreover, the low levels of DOAC adherence also suggested that Vietnamese patients might not be able to afford the long-term use of DOAC owing to much higher costs or the non-monitoring DOAC might lower patient awareness of anticoagulants [16].

By excluding patients with heart valve replacement in the sensitivity analysis, we found that levels of medication knowledge and adherence were similar between patients taking VKA and DOAC, regardless of their VTE risks. This also strengthens our findings and differentiates the anticoagulation practices in high- and low-resource countries. In the former settings, both VKA and DOAC can be accessible and affordable [17]. However, in Vietnam or other Southeast Asian countries, costs could act as a deterrent to DOAC. With less access or affordability to DOAC, physicians tend to have more concerns over patient adherence to VKA because of complicated monitoring and uses. This might not be the case in Vietnam because our findings showed no significant difference between patients taking DOAC and VKA. With the current evidence, to avoid health care burdens in low- and middle-income countries, patients may not need to take the DOAC regimens if no additional benefits are justified.

To the best of our knowledge, this study is one of the first to investigate the patients’ knowledge and adherence to both VKA and DOAC in Southeast Asian settings. In addition, our study is also among the first to identify factors associated with the adherence level (high, medium, or low) to oral anticoagulants. However, there are some limitations to our study. First, we could not examine the association between knowledge or adherence and the effectiveness of oral anticoagulation therapy. Second, we lacked data about illiterate patients and other potential confounders, eg, lifestyles, diets, concomitant drugs, etc., which could introduce some biases into the study. Third, we did not get access to the MMAS-8 [15]. This might result in a reduced internal consistency of the scale when assessing medication adherence. Moreover, our sample size was fairly small to draw any strong conclusions about the moderating factors of patients’ knowledge or medication adherence.

## 5 Conclusion

In Southeast Asian clinical practice, the knowledge and high adherence of patients taking oral anticoagulants remain limited. Further studies in similar settings should examine the associations between these factors and the effectiveness of the regimens on patients. More effective measures on moderating factors of knowledge and adherence should also be developed and implemented to optimize the anticoagulation therapy, improve the patients’ health outcomes, and mitigate the associated adverse effects.

## Funding

This research was funded by 10.13039/100017047Nguyen Tat Thanh University, Ho Chi Minh City, Vietnam.
