# Supplementary material for: Knowledge and medication adherence of oral anticoagulant-taking patients in Vietnam
Source: Res Pract Thromb Haemost. 2023 Jan 11;7(1):100044. doi: 10.1016/j.rpth.2023.100044 (PMC9932105; doi:10.1016/j.rpth.2023.100044)
Supplement: Supplementary Material [file mmc1.docx]

**Knowledge and medication adherence of oral anticoagulant-taking patients in vietnam**

Minh-Hoang Tran^1^, Hoang Hai Nguyen^2^, Quoc Khanh Mai^3^, Hong Tham Pham^1,3^

^1^Department of Pharmacy, Nhan Dan Gia Dinh Hospital, Ho Chi Minh City, Vietnam

^2^Department of Cardiology, Nhan Dan Gia Dinh Hospital, Ho Chi Minh City, Vietnam

^3^Department of Pharmacy, Nguyen Tat Thanh University, Ho Chi Minh City, Vietnam

**Supplementary Checklist.** STROBE Statement—Checklist of items that should be included in reports of ***cross-sectional studies***

|  | Item No | Recommendation |
| --- | --- | --- |
| **Title and abstract** | 1 | (*a*) Indicate the study’s design with a commonly used term in the title or the abstract  **Abstract** |
|  |  | (*b*) Provide in the abstract an informative and balanced summary of what was done and what was found  **Abstract** |
| Introduction | | |
| Background/rationale | 2 | Explain the scientific background and rationale for the investigation being reported  **Introduction (paragraph 1, 2, and 3)** |
| Objectives | 3 | State specific objectives, including any prespecified hypotheses  **Introduction (paragraph 3)** |
| Methods | | |
| Study design | 4 | Present key elements of study design early in the paper  **Methods (section 2.1)** |
| Setting | 5 | Describe the setting, locations, and relevant dates, including periods of recruitment, exposure, follow-up, and data collection  **Methods (section 2.1)** |
| Participants | 6 | (*a*) Give the eligibility criteria, and the sources and methods of selection of participants  **Methods (section 2.1)** |
| Variables | 7 | Clearly define all outcomes, exposures, predictors, potential confounders, and effect modifiers. Give diagnostic criteria, if applicable  **Methods (section 2.2 and 2.4)** |
| Data sources/ measurement | 8* | For each variable of interest, give sources of data and details of methods of assessment (measurement). Describe comparability of assessment methods if there is more than one group  **Methods (section 2.1)** |
| Bias | 9 | Describe any efforts to address potential sources of bias  **Methods (section 2.4 and 2.5), Supporting Information (Supplementary Table S1)** |
| Study size | 10 | Explain how the study size was arrived at  **Methods (section 2.3)** |
| Quantitative variables | 11 | Explain how quantitative variables were handled in the analyses. If applicable, describe which groupings were chosen and why  **Methods (section 2.5)** |
| Statistical methods | 12 | (*a*) Describe all statistical methods, including those used to control for confounding  **Methods (section 2.5)** |
|  |  | (*b*) Describe any methods used to examine subgroups and interactions  **Methods (section 2.5)** |
|  |  | (*c*) Explain how missing data were addressed  **Methods (section 2.5)** |
|  |  | (*d*) If applicable, describe analytical methods taking account of sampling strategy  **Methods (section 2.1)** |
|  |  | (*e*) Describe any sensitivity analyses  **NA** |
| Results | | |
| Participants | 13* | (a) Report numbers of individuals at each stage of study—eg numbers potentially eligible, examined for eligibility, confirmed eligible, included in the study, completing follow-up, and analysed  **Supporting Information (Supplementary Figure S1)** |
|  |  | (b) Give reasons for non-participation at each stage  **Supporting Information (Supplementary Figure S1)** |
|  |  | (c) Consider use of a flow diagram  **Supporting Information (Supplementary Figure S1)** |
| Descriptive data | 14* | (a) Give characteristics of study participants (eg demographic, clinical, social) and information on exposures and potential confounders  **Results (Table 1)** |
|  |  | (b) Indicate number of participants with missing data for each variable of interest  **Supporting Information (Supplementary Table S1)** |
| Outcome data | 15* | Report numbers of outcome events or summary measures  **Results (Table 2 and Table 4)** |
| Main results | 16 | (*a*) Give unadjusted estimates and, if applicable, confounder-adjusted estimates and their precision (eg, 95% confidence interval). Make clear which confounders were adjusted for and why they were included  **Results (Table 3 and Table 5)** |
|  |  | (*b*) Report category boundaries when continuous variables were categorized  **Results (Table 1)** |
|  |  | (*c*) If relevant, consider translating estimates of relative risk into absolute risk for a meaningful time period  **NA** |
| Other analyses | 17 | Report other analyses done—eg analyses of subgroups and interactions, and sensitivity analyses  **Methods (section 2.5)** |
| Discussion | | |
| Key results | 18 | Summarise key results with reference to study objectives  **Discussion (paragraph 1)** |
| Limitations | 19 | Discuss limitations of the study, taking into account sources of potential bias or imprecision. Discuss both direction and magnitude of any potential bias  **Discussion (paragraph 6)** |
| Interpretation | 20 | Give a cautious overall interpretation of results considering objectives, limitations, multiplicity of analyses, results from similar studies, and other relevant evidence  **Discussion (paragraph 2–5)** |
| Generalisability | 21 | Discuss the generalisability (external validity) of the study results  **Discussion (paragraph 3–5)** |
| Other information | | |
| Funding | 22 | Give the source of funding and the role of the funders for the present study and, if applicable, for the original study on which the present article is based **Funding** |

*Give information separately for exposed and unexposed groups.

**Note:** An Explanation and Elaboration article discusses each checklist item and gives methodological background and published examples of transparent reporting. The STROBE checklist is best used in conjunction with this article (freely available on the Web sites of PLoS Medicine at http://www.plosmedicine.org/, Annals of Internal Medicine at http://www.annals.org/, and Epidemiology at http://www.epidem.com/). Information on the STROBE Initiative is available at www.strobe-statement.org.

**
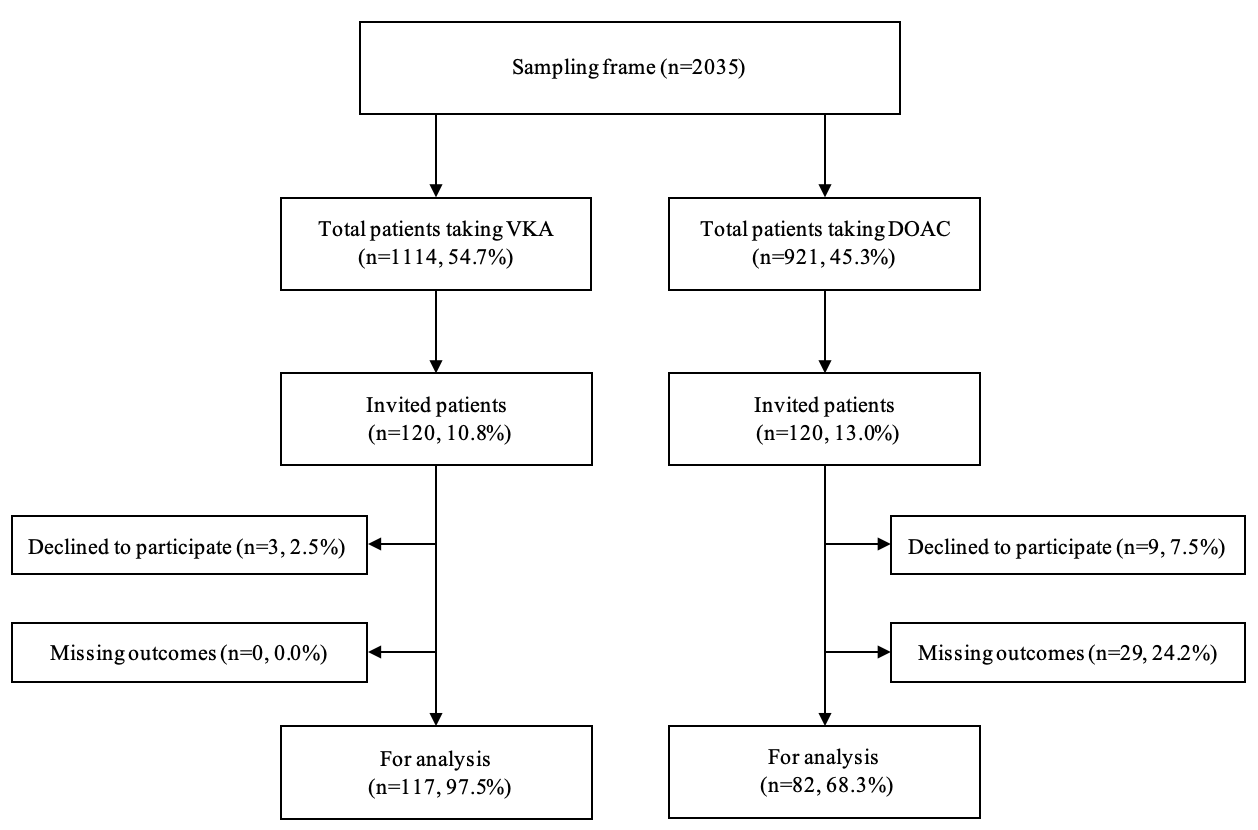
**

**Supplementary Figure S1.** Sample selection flowchart.

Abbreviations: DOAC: direact-acting oral anticoagulants; VKA: vitamin K antagonists.

Note: Among patients taking DOAC, 29 refused to answer or disclose relevant information to measure the study outcomes, leading to the missing proportion of 24.2%.

**Supplementary Table S1.** Characteristics of the participants included and excluded from the analyses

| Characteristics | VKA | | |  | DOAC | | |
| --- | --- | --- | --- | --- | --- | --- | --- |
|  | Included  (n=117) | Excluded  (n=3) | p-value^a^ |  | Included  (n=82) | Excluded  (n=38) | p-value^a^ |
| Interviewed subjects, n (%) |  |  | 0.313 |  |  |  | 0.857 |
| Patients | 104 (88.9) | 2 (66.7) |  |  | 68 (82.9) | 31 (81.6) |  |
| Direct caretakers | 13 (11.1) | 1 (33.3) |  |  | 14 (17.1) | 7 (18.4) |  |
| Age, median (Q1–Q3) | 60 (51–69) | 58 (49–62) | 0.614 |  | 67 (60.3–72.8) | 61 (54–69) | 0.101 |
| Age category, n (%) |  |  | 0.819 |  |  |  | 0.186 |
| 18–65 years old | 85 (72.6) | 2 (66.7) |  |  | 43 (52.4) | 15 (39.5) |  |
| > 65 years old | 32 (27.4) | 1 (33.3) |  |  | 39 (47.6) | 23 (60.5) |  |
| Gender, n (%) |  |  | 0.583 |  |  |  | 0.363 |
| Male | 51 (43.6) | 2 (66.7) |  |  | 44 (53.7) | 17 (44.7) |  |
| Female | 66 (56.4) | 1 (33.3) |  |  | 38 (46.3) | 21 (55.3) |  |
| Education, n (%) |  |  | 0.553 |  |  |  | 0.688 |
| Primary or lower | 36 (30.8) | 0 (0.0) |  |  | 20 (24.4) | 8 (21.1) |  |
| Secondary or higher | 81 (69.2) | 3 (100.0) |  |  | 62 (75.6) | 30 (78.9) |  |
| Occupational status, n (%) |  |  | 0.193 |  |  |  | 0.513 |
| High | 8 (6.8) | 0 (0.0) |  |  | 4 (4.9) | 2 (5.3) |  |
| Middle | 24 (20.5) | 2 (66.7) |  |  | 11 (13.4) | 8 (21.1) |  |
| Low | 85 (72.6) | 1 (33.3) |  |  | 67 (81.7) | 28 (73.7) |  |
| Previous counseling, n (%) | 20 (17.1) | 1 (33.3) | 0.442 |  | 14 (17.1) | 9 (23.7) | 0.392 |
| Previous VTE, n (%) | 9 (7.7) | 0 (0.0) | 0.617 |  | 13 (15.9) | 6 (15.8) | 0.993 |
| Abbreviations: DOAC: direact-acting oral anticoagulants; Q1–Q3: interquartile range; VKA: vitamin K antagonists; VTE: venous thromboembolism.  ^a^p-values were calculated using Chi-Square/Fisher test for categorical variables and Mann–Whitney test for continuous variables.  Note: Percentages may not add up to 100 due to rounding. | | | | | | | |

**Supplementary Table S2.** Correct responses to the adjusted AKT questionnaire

| Items, n (%) | VKA  (n=117) | DOAC  (n=82) |
| --- | --- | --- |
| **Section A** | | |
| 1. What is the name of your anticoagulant medicine? | 100 (85.5) | 59 (72.0) |
| 2. Why has your doctor prescribed you this medicine? | 82 (70.1) | 49 (59.8) |
| 3. How does this medicine work in your body? | 45 (38.5) | 27 (32.9) |
| 4. How many times a day do you need to take this medicine? | 102 (87.2) | 61 (74.4) |
| 5. Why is it important to take this medicine exactly as your doctor has told you? | 67 (57.3) | 30 (36.6) |
| 6. Is it important to take this medicine at the same time each day? | 105 (89.7) | 57 (69.5) |
| 7. Is it okay to double the next dose of this medicine if you miss a dose? | 103 (88.0) | 80 (97.6) |
| 8. Is it appropriate to stop taking this medicine once you feel better? | 107 (91.5) | 74 (90.2) |
| 9. Is it safe to take anti-inflammatory medicines like ibuprofen (Nurofen® or Advil®) while you are taking this medicine? | 72 (61.5) | 47 (57.3) |
| 10. Will drinking too much alcohol increase the risk of side effects with this medicine? | 73 (62.4) | 55 (67.1) |
| 11. Would you inform a surgeon, dentist or other health professional that you are taking this medicine before undergoing surgery or a procedure? | 103 (88.0) | 65 (79.3) |
| 12. Is it important that all the health care practitioners you see know that you are taking this medicine? | 103 (88.0) | 66 (80.5) |
| 13. What is the most important side effect of this medicine? | 65 (55.6) | 28 (34.1) |
| 14. THREE signs of side effects that you should watch out for while taking this medicine are: | 67 (57.3) | 28 (34.1) |
| 15. THREE things you can do to reduce your risk of side effects are: | 27 (21.4) | 19 (23.2) |
| 16. What is the best step to take if you accidentally take too much of this medicine? | 29 (24.8) | 14 (17.1) |
| **Section B** | | |
| 1. What is your target INR range? | 32 (27.4) | – |
| 2. What was your last INR reading? | 76 (65.0) | – |
| 3. Are regular INR tests necessary to know how well this medicine is working? | 100 (85.5) | – |
| 4a. Is it possible for what you eat to affect your warfarin therapy? | 76 (65.0) | – |
| 4b. If you answered ‘Yes’ above, list THREE foods that can affect your anticoagulant therapy. | 76 (65.0) | – |
| 5. List one vitamin that can significantly affect your anticoagulant therapy. | 12 (10.3) | – |

**Supplementary Table S3.** “No” responses to the MGLS questionnaire

| Items, n (%) | VKA  (n=117) | DOAC  (n=82) |
| --- | --- | --- |
| 1. Do you ever forget to take your medicine? | 70 (59.8) | 57 (69.5) |
| 2. Are you careless at times about taking your medicine? | 78 (66.7) | 46 (56.1) |
| 3. When you feel better do you sometimes stop taking your medicine? | 109 (93.2) | 77 (93.9) |
| 4. Sometimes if you feel worse when you take the medicine, do you stop taking it? | 95 (81.2) | 64 (78.1) |
